# Predicting women’s career decisiveness in the ICT sector: A serial multiple mediation model among MIS students

**DOI:** 10.1371/journal.pone.0316154

**Published:** 2024-12-20

**Authors:** Nuray Akar, Tayfun Yörük, Ömür Tosun

**Affiliations:** Department of Management Information Systems, Akdeniz University, Antalya, Turkey; Kathmandu Model College, Tribhuvan University, NEPAL

## Abstract

This study explores the factors that influence women’s career decisions in the Information and Communication Technologies (ICT) sector, as perceived by women Management Information Systems (MIS) students. It examines how career optimism (CO) and perceived employability (PE) affect the link between irrational beliefs about employment (IB) and career decisiveness (CD). The study involved 232 female students from ICT-related programs in Turkey. Data were collected using four different scales and analyzed for accuracy and connections between factors. To prevent the effects of common method bias, Harman’s single-factor test was used, followed by an analysis of mediation effects. Results showed that women students’ IB about employment negatively impacted their CD. CO helped mediate this effect. Moreover, both CO and PE together had a mediating role in how IB affected CD. These findings offer valuable insights into the individual and contextual factors shaping women’s career decisions. They also support initiatives to boost women’s career stability, which aligns with the United Nations (UN) Sustainable Development Goals (SDGs) 5 and 8. Encouraging career optimism and employability may reduce the negative effects of IB and contribute to a fairer job market where women can pursue ambitious careers.

## Introduction

Equal access to employment for women is a priority on the United Nations’ (UN) sustainable development agenda. It is of particular importance to achieve the targets set out in Goal 5: “Achieve gender equality and empower all women and girls”, as this will facilitate the realization of the full human potential in sustainable development. Goal 8: “Promote sustained, inclusive and sustainable economic growth, full and productive employment and decent work for all”, envisages the construction of more sustainable and people-centered economies through the improvement of the employment conditions of youth and women in particular [1]. However, the International Labor Organization (ILO) highlighted a new indicator called ‘jobs gap’ in its March 2023 report, which reveals that progress in reducing gender imbalances in access to employment and working conditions has been very slow over the last 20 years (2005–2022). According to this indicator, 15% of working-age women worldwide are unable to get a job even though they want to work. The jobs gap is an indicator that measures labor underutilization beyond unemployment. It encompasses all individuals who are actively seeking employment but are currently not employed. In this regard, it serves as a valuable complement to the unemployment rate. It is an essential indicator for evaluating the challenges faced by women in securing employment. The long-term trajectory of gender disparity in the jobs gap represents a persistent challenge for the labor market [2]. The 2024 Global Gender Gap Report of the World Economic Forum (WEF), which is based on the index results of 146 countries, indicates that global progress in terms of gender equality is relatively slow. Based on the current rate of progress, it is estimated that it will take 134 years to achieve global gender parity. In terms of the subindexes, it will take 20 years to close the gender gap in educational attainment. With regard to the economic participation and opportunity subindex, which encompasses five indicators, namely the labor-force participation rate, wage equality for similar work, estimated earned income, legislators, senior officials and managers, and professional and technical workers, the estimated period required for full parity is 152 years [3].

The "5.5.2 Proportion of women in managerial positions" indicator is an essential metric for evaluating the progress made in achieving gender equality in the workplace and its implications on sustainable development. The gender gap in leadership positions is a significant factor contributing to the persistent disparities in gender equality [4]. As indicated in the UN Statistics Division’s Sustainable Development Goals Report for 2024, the proportion of women occupying managerial positions remains unchanged from 2016 at 27.5 per cent, despite women comprising 40 per cent of global employment in 2022. Based on the observed rate of change, it is estimated that achieving equality in managerial roles will take approximately 176 years [5]. Furthermore, the 2023 assessment conducted by UN Women indicates that women remain underrepresented in managerial roles within the workplace. Currently, only 28.2% of managerial positions in the global business sector are held by women [4].

Another crucial objective for achieving sustainable development through employment equity is to attain the goal set forth in paragraph 8.5 of the Sustainable Development Goals (SDGs), which states that "By 2030, achieve full and productive employment and decent work for all women and men, including for young people and persons with disabilities, and equal pay for work of equal value." [1]. As indicated by the ILO’s 2022 Jobs Gap Indicator, 15% of working-age women globally are unable to secure employment, despite their desire to work. In 2023, the discrepancy in employment opportunities between women and men is estimated at 220.7 million and 214.1 million, respectively. In other words, while the gender jobs gap in 2023 is 13.7 per cent, this rate is 9.3 per cent for men [6]. Furthermore, the UN Statistics Division highlights that by 2023, the proportion of young women who are neither in education nor in employment is approximately twice that of young men globally [5].

One of the key indicators that presents a significant challenge to achieving Target 8.5 and, subsequently, sustainable development is the gender pay gap, which represents one of the most pervasive forms of social injustice. In calculating this discrepancy, the ILO employs the methodology of averaging hourly wages, as recommended by the sustainable development indicator "8.5.1 Average hourly earnings of female and male employees". This provides a global analysis of the gender pay gap, comparing the average hourly earnings of male and female workers to assess progress made towards sustainable development. The ILO’s 2018–2019 Global Wage Report, based on data from 73 countries covering approximately 80 per cent of workers globally, states that the weighted global gender pay gap is approximately 16 per cent [7]. The impact of the global pandemic and the subsequent cost of living crisis on this gender pay gap, which is based on data from 2014 to 2016, is also detailed in the International Labor Organization’s (ILO) Global Wage Report 2022–2023. This updated report reflects changes in factor-weighted gender pay gaps between 2019 and 2021 (or 2022) in a smaller sample of 22 countries. The report demonstrates that the gender pay gap is positive in all 22 countries that were subjected to analysis. In other words, the gender pay gap persists in labor markets around the world, with women being remunerated at a lower rate than men on average. An analysis of the gender pay gap over time reveals that there has been no significant change since the period preceding the onset of the global pandemic caused by the SARS-CoV-2 virus. A review of the period following the onset of the pandemic reveals a paucity of notable shifts, with minimal variation observed between 2019 and 2021–2022 [8].

On the other hand, according to the European Institute for Gender Equality (EIGE), the increasing need for Science, Technology, Engineering, Mathematics (STEM) and Information and Communication Technologies (ICT) skills in almost all sectors, coupled with digitalization, presents a great opportunity for greater inclusion of women in employment. Despite this, only 17 percent of the nearly 8 million ICT professionals in Europe are women [9]. The 2022 DESI report [10] highlights that women make up only one-third of STEM graduates and one-fifth of ICT graduates [10]. Gender stereotypes that suggest technology is a male-dominated field are prevalent in many contexts. These and other gender biases can negatively impact women’s confidence in their digital skills and influence their educational choices. Ultimately, such biases create barriers to entry for women in these fields [11]. It is erroneous to assume that an increase in the number of women pursuing and continuing to engage in STEM education will inevitably result in their long-term participation and representation in the labor force within this field [12]. As indicated in the UN Women’s Gender Equality 2023 Status Report, the participation of women in STEM fields remains significantly below that of their male counterparts. Indeed, women occupy less than 25% of jobs in STEM and ICT fields across the globe [4].

Furthermore, the 2023 Global Gender Gap Report published by the World Economic Forum (WEF) emphasizes the striking underrepresentation of women in the science, technology, engineering and mathematics (STEM) workforce. Furthermore, it is observed that there is a discernible reduction in the proportion of women remaining in the labor market, even within the first year of graduation, in STEM-related fields. Conversely, while 29.4% of those entering the field of STEM are women, this proportion declines at the senior management level. The proportion of women in senior management positions within the STEM sector is 17.8 per cent, while the figure for senior leadership roles is 12.4 per cent [13]. EIGE [14] additionally observes that women’s monthly earnings in the ICT sector are inferior to those of men, a disparity that can be attributed to gender-based disparities in average working hours, positions, and hourly wages. The European Parliamentary Research Service (EPRS) indicates that the discrepancy in earnings between women and men in the ICT sector is approximately 20%. The data set, sourced from Statista and spanning the period from 2019 to 2022, comprises responses from 120,000 individuals in Canada, Ireland, the United Kingdom, and the United States who were seeking employment in the technology sector during the aforementioned timeframe. The findings indicate that, in 2022, women applying for the same position in the same company in the technology sector will be offered an average salary that is 3.5 per cent less than that offered to men [15].

While the technological skills requirement, which has emerged as a prominent feature of the labor market in the context of digitalization, presents a significant opportunity for enhancing women’s employment prospects, it cannot be viewed as a standalone solution for achieving full human potential in the pursuit of sustainable development. Inequalities in terms of women’s participation in STEM education, graduation from this field, employment in fields such as ICT and career determination in the field remain. Prior research has also indicated that pivotal junctures in the academic and professional advancement of women in STEM fields, such as university admission, integration into the labor market, and promotion within their chosen fields, are marked by a notable decline in female participation [16, 17]. Furthermore, it has been demonstrated that despite holding a STEM degree, women are less likely to secure positions in these fields [18, 19]. Additionally, there is a notable underrepresentation of women in the STEM workforce [20–22]. It is also emphasized that women in STEM fields often tend to leave this field and switch to career paths in other fields [23–25].

At this point, Kuchumova et al. [26] draw attention to the fact that women’s underrepresentation in traditionally male-dominated jobs is a comprehensive phenomenon triggered by individual, organizational, and socio-structural factors, rather than a narrow perspective that attributes women’s underrepresentation in traditionally male-dominated jobs solely to a lack of necessary knowledge, skills, and dispositions. Naseviciute and Juceviciene [27] also reveal that there are various intrapersonal and environmental barriers to women’s advancement to senior leadership positions in ICT. The key intrapersonal barriers include the lack of belonging, lack of self-confidence, concerns about work-life balance, and perceived glass ceiling. The primary environmental barriers can be attributed to a number of factors, including male domination, the prevalence of socially gendered professions, pervasive stereotypes about gender, unconscious gender bias, a dearth of female role models, and pervasive stereotypes about ICT businesses. Cultural definitions of gender that favor masculine skills and abilities in STEM roles have an impact on women’s career choices in this field [21].

The reflections of traditional gender ideology, which prioritizes family responsibilities and motherhood roles, manifest themselves as discriminatory organizational practices in the recruitment and remuneration of women in STEM roles [19]. The stereotypical beliefs about the STEM field have been found to have a negative impact on self-efficacy towards STEM jobs and outcome expectations related to STEM careers [28]. A number of factors, including self-perception, self-efficacy, interests and family commitment, play a significant role in the decision-making and permanence stages of STEM careers [17]. In light of the pivotal role of innovation and technology in the contemporary global economy, the issue of turnover in STEM fields has emerged as a significant concern [20]. While prior studies have addressed women’s retention in STEM (e.g., [12, 26, 29]), it is notable that the relevant literature focuses more on persistence in STEM education (e.g., [17, 30, 31]). At this juncture, there is a significant gap in the existing literature with regard to the empirical revelation of the theoretical underpinnings of the chain of relationships between the factors that encourage or hinder women’s engagement and persistence in this specific field after graduation. This is particularly evident in the absence of context-specific data provided by women on the basis of career choice focused on a specific field of education and a related sector.

The present research examines the factors affecting women’s career decisiveness (CD) in the ICT sector, focusing on women Management Information Systems (MIS) students. In this respect, the serial multiple mediation effect of career optimism (CO) and perceived employability (PE) on the effect of irrational beliefs about employment (IB) on CD was analyzed. The research model is based on Lent, *et al*. [32]‘s Social Cognitive Career Theory (SCCT) and Ford [33]‘s Motivational Systems Theory (MST) to explain the interactions among the variables. Through the aforementioned research model, whose conceptual and theoretical foundations are detailed in the following sections, firstly, the total effect of women MIS students’ IBs on CD was analyzed. Then, it is tested whether IB has a serial indirect effect on CD through CO and PE. Thus, the chain of relationships among the factors affecting women’s CD in the ICT sector has been revealed and contributed to the body of knowledge in the related literature. This chain of relationships is expected to guide the development of comprehensive insights into the factors influencing women’s career decisions in technology-oriented fields. The research results could inform policies and practices aimed at promoting diversity and equity in the workforce.

In summary, this study aims to provide a comprehensive understanding of the dynamics affecting women’s employment and to suggest ways to increase women’s participation and contribution to the economy. In this context, integrating major theories that guide a better understanding of the multidimensional effects on career decision-making is based on. Thus, complementary perspectives considering the critical role of cognitive, emotional, and environmental factors provide a richer understanding of the career development process. This framework focuses on understanding women’s career behaviors in a traditionally male-dominated sector such as ICT. The research examines the interactions of social and cognitive factors influencing the career decisions of female MIS students and their motivations for career decisiveness. In this direction, the main research question was designed to analyze the impact of female MIS students’ employment-related IBs on their CDs in the ICT sector. The research model was built on how CO and PE mediate this relationship. As a result, insights are provided into the interactions of individual and contextual factors in predicting women’s career decisiveness in the ICT sector. The motivating or constraining effects of cognitive beliefs, cultural stereotypes, and social structures have been contextually explored to support women’s decisiveness and continuity in specific career fields.

### Contextual framework

The research was conducted with female students enrolled in the MIS department of a state university in Turkey. The department offers a four-year formal undergraduate education. The department comprises the disciplines of information management, decision sciences and information technologies. The department’s curriculum mandates that Career Planning be taken as a compulsory course during the first semester. In the final semester, a Workplace Training course is offered to provide students with the opportunity to apply theoretical knowledge, to train qualified candidates and to increase the employment rate of graduates. This course involves full-time practical training in an enterprise. The department also offers postgraduate programs leading to the degrees of Master of Science and Doctor of Philosophy, with the requirement of a thesis [34]. In accordance with the 2023–2024 academic year’s higher education statistics, the department has a female student population comprising 31.8% of the total enrolment [35].

Turkey is positioned at 127th place among 146 countries, with a score of 64.5% on the Global Gender Gap Index for 2024. A review of the sub-indices of the Index reveals that only 34.6% of graduates in STEM fields are women in Turkey, which is positioned at 90th place in the educational attainment sub-index. Turkey’s ranking in the economic participation and opportunity subindex is 133rd. With regard to the specific indicators that comprise the subindex, Turkey is positioned at 132nd in terms of labor-force participation rate, 96th in wage equality for similar work, 121st in estimated earned income, 124th in legislators, senior officials and managers, and 101st in professional and technical workers. The labor force participation rate of women in Turkey is 35.1%, which is below the global average. There is a discrepancy of 24.9 percentage points between the estimated earned income of men and women in Turkey. While the representation of women in senior management positions in Turkey is 18.4 per cent, 42.4 per cent of those working in professional and technical positions are women [3]. Despite the expansion of the ICT sector in Turkey in terms of employment, the representation of women in the sector remains comparatively low. In 2023, the proportion of women employed in the ICT sector in Turkey increased by 11% to 237,000, representing 31% of the total workforce [36].

One of the key objectives of Turkey’s twelfth development plan, spanning the period 2024–2028, is to enhance the participation of women in STEM fields, where their representation remains relatively low [37]. Nevertheless, as is the case in numerous other countries, female students in Turkey often lack the requisite motivation to pursue further studies in STEM subjects and to embark upon a career in this field. The underlying cause of this lack of motivation can be attributed to gender biases and socialization processes that culturally promote male dominance and female submissiveness [38]. In Turkey, as in other developing countries, the assumption that certain sectors and jobs are more suitable for men presents a significant barrier to women’s employment in certain fields and professions, as well as their advancement in their careers. This socio-cultural environment, which plays a significant role in perpetuating the gender gap, results in a lack of self-confidence among women in STEM fields. Furthermore, the prioritization of family and domestic responsibilities for many women constrains their capacity to compete with men in the workplace. The unfavorable working conditions prevalent in the IT sector, including lengthy working hours and wage disparities between men and women, impede women’s pursuit of a harmonious work-life balance and access to flexible work arrangements with equitable terms. Furthermore, women are perceived as unsuitable for top management positions due to the assumption that the characteristics associated with management are less aligned with their personalities. This perception is often reinforced by the idea that women are perceived as more emotional individuals [39].

The gender-based division of labor in the IT sector has resulted in the attribution of hard skills to men and soft skills to women. This approach results in female employees in the sector being assigned tasks that are perceived to be more socially oriented, such as project and quality management. In contrast, tasks that are believed to require more mental skills, such as system analysts and computer technology-based computing and engineering jobs, are typically entrusted to men [40]. Consequently, it is evident that women in the ICT sector are more inclined towards support roles, whereas areas such as project management have witnessed an increase in female representation at the managerial level. Conversely, domains such as software and engineering remain predominantly male-dominated [41]. The lack of opportunities to interact with female role models and mentors represents a significant barrier to the development of a female identity in STEM [42].

The continued inequality in women’s access to technology results in a loss of opportunity to develop the new skills required for digital transformation. Furthermore, the representation of women in the ICT sector remains low. Consequently, women are unable to reap the benefits of digital-intensive sectors, where the demand for technological expertise has grown, particularly in the context of the pandemic. This is especially pertinent in terms of increasing the representation of women in gainful employment and reducing the gender pay gap [43]. The gender regime in Turkey, which dictates that women should work in ’appropriate’ environments and in ’appropriate’ ways, has a significant impact on women’s career choices, influencing them to pursue roles that are perceived as more ’sterile’, ’close to home’, and away from the factory and its masculine character. Additionally, these roles are often not perceived as exerting pressure on the home-work balance. Consequently, it is evident that women predominantly gain experience in formal sectors and white-collar occupations following graduation and prior to marriage or childbirth. Furthermore, even professional women exit the labor market in subsequent years due to prevailing gender dynamics [44].

In order to gain a deeper understanding of the factors influencing women’s involvement and career progression in the ICT field following graduation from university, it is essential to examine the complex interrelationships between these variables. This study aims to expand the existing literature on gender equality in the context of the ICT sector in Turkey, with a specific focus on women’s career stability in the sector. In this context, the relationships between women’s irrational beliefs about employment, career optimism attitudes, employability perceptions and career determination in the context of sexist structures in national and organizational culture are examined in the ICT sector. No previous study has addressed these contextual dynamics in the relevant literature. The encouragement of women in Turkey to demonstrate career determination in the ICT sector is a strategy that can facilitate the achievement of the national development goal of increasing the proportion of women in STEM fields. In light of the aforementioned, this study offers invaluable insights for relevant actors.

### Conceptual framework

The literature uses the terms career decisiveness, decidedness, certainty, and persistence interchangeably for CD [45], which refers to an individual’s certainty in their career choice. In this study, we consider the related concept as career decisiveness (CD). This study is based on SCCT [32], which focuses on women’s CD in the ICT sector. SCCT, which originates from Bandura [46]‘s Social Cognitive Theory (SCT), proposes that career decision-making factors are variable and dynamic. SCCT addresses individual and environmental factors in career decision-making. The theory focuses on cognitive-personal variables, such as self-efficacy and outcome expectations, and their interaction with environmental variables, such as gender and social barriers. Thus, it provides a framework for the career entry process of late adolescents. It illuminates students’ career decisions, including their interests, career preferences, and educational pursuits. In this respect, it provides a basis for the present research that enables the development of insights into women students’ CDs in the ICT sector. In this study, Ford [33]‘s MST is another theoretical framework that is taken as a basis for expanding the perspective on CD. MST comprehensively integrates the theoretical and empirical aspects of different theories of motivation. In this framework, it conceptualizes motivation as an interaction between personal goals, capability beliefs, context beliefs, and emotional arousal processes. According to Chatterjee, *et al*. [47], motivational factors play a significant role in CD. Therefore, MST provides a theoretical foundation for individuals to explore themselves and their environment when making career choices based on their desired outcomes, perceptions of their skills, perception of social support, and emotions that enable them to stimulate their energy [48].

These two theoretical bases for explaining CD are based on individual and environmental factors. However, many studies only focus on the cognitive-personal variables of SCCT [49]. In this context, the career choice and development process are dominated by self-efficacy in SCCT. However, individuals may not always have an active role in the CD process. Issues beyond the voluntary control of the individual through knowledge schemas and stereotypes may also be influential in this process. According to MST, motivational factors collectively influence behavior, but independently of each other. Thus, it also sheds light on the aspects in which the interaction of goals, beliefs, and emotions in career orientation may occur beyond the control of the individual [48]. In his study updating SCCT from the perspective of career development and counselling, Lent [50] emphasizes that socially constructed factors, especially gender, can affect career decisions. The development of cognitive-personal variables such as self-efficacy and outcome expectations depends on the individual’s socialization dynamics. Environmental variables such as gender and social barriers can affect career opportunities by limiting the individual’s career interests and shape career goals based on contextual conditions.

For example, there are stereotypes that associate women more with social work and men more with mathematics and science, depending on their interests and compassion. Although these stereotypes reflect horizontal discrimination in the labor market, they ultimately pave the way for women and men to pursue career fields that are compatible with them. On the other hand, stereotypes about men being more ambitious and intelligent than women reflect vertical discrimination in the labor market. It therefore provides a basis for men to occupy leadership positions. The fact that women are still under-represented in STEM education and employment is therefore not only a result of their individual preferences. Factors such as cultural stereotypes, the attitudes of parents, teachers and peers shape women’s self-perceptions of their abilities, achievements, and career prospects. All these cumulative effects act as a barrier to women’s interest and integration in STEM and to their progress in this field [51].

**Fig 1:**
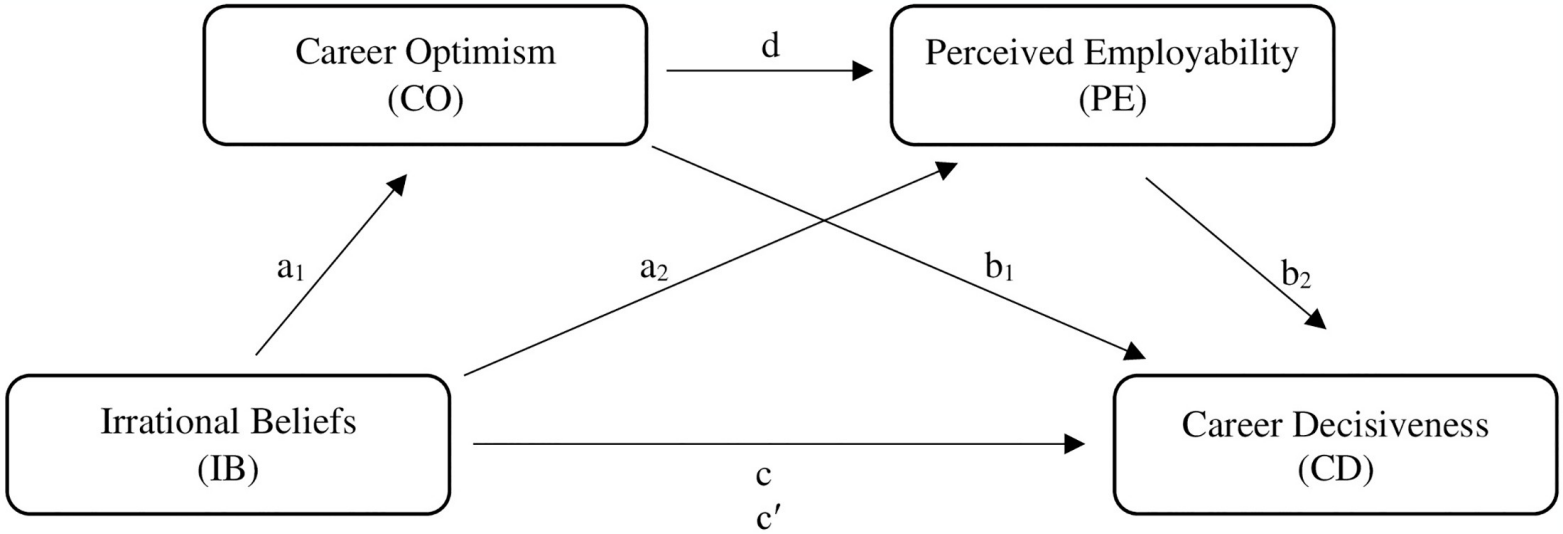
Multiple serial mediation model of the study.

Based on this theoretical framework, the current study, which focuses on the chain of relationships among the factors affecting the CD of women MIS students in the ICT sector, considers employment-related IB as the predictor variable. IB, as a dimension reflecting career anxiety, refers to the difficulties that university graduates may face when entering the labor market [52]. IBs related to CD include self-efficacy beliefs, negative emotions, mixed thoughts and attachment anxiety [53]. IBs that indicate misunderstandings, misinterpretations, false assumptions, and false expectations about career have a negative impact on career decisions [54]. Another variable included in the research is CO. CO refers to an individual’s tendency to think positively about their career [55]. CO enables individuals to see the obstacles to their career as surmountable. CO enables individuals to see the negativity towards their career as a temporary situation. CO gives individuals the strength to fight for their careers despite the setbacks they face [56]. The final variable in the research model is PE. PE reflects individuals’ perceptions of their competitiveness in the labor market in the context of their own skills and experience [57]. PE involves individual perceptions of the likelihood of entering and remaining in employment. It therefore reflects a subjective assessment. It is related to probabilities based on personal and structural factors. It therefore depends on the possibilities of integrating individual, work-related, organizational, and social interactions. As such, it does not consist solely of an assessment of self-efficacy [58]. The proposed research model is as shown in Fig 1.

### The relationship between IB and CD

Irrational beliefs about employment (IB) is one of the main challenges in making a sustainable career decision [59]. Issues such as perfectionism, external control, false inferences, generalizations, and self-esteem are within the scope of IBs related to students’ career choices. These beliefs create difficulties in the CD [60]. Students who are undecided about their career may experience career anxiety. This anxiety includes issues such as personal ability, IBs, vocational education practice and the labor market [61]. There are strong but invisible barriers that limit women’s career opportunities, particularly in STEM, due to cultural beliefs about gender and stereotypes that favor men [62].

The IBs that affect women students’ CD towards STEM include not seeing themselves as suitable for this field in terms of gender roles, perceiving themselves as inadequate in this field, and dysfunctional thoughts about career choice [63]. Based on this background, the first hypothesis of the study is proposed as follows:

*H*_*1*_: *Women MIS students’ employment related IBs have a negative and significant effect on women’s CDs in the ICT sector*.

### Mediating role of CO

Career optimism (CO), which reflects an attitude, acts as a bridge between different individual components of MST and CD. In this context, CO mediates the relationship between MST components such as ability beliefs, contextual beliefs and emotions and CD [48]. CO contributes to the development of positive career attitudes such as CD [64, 65] and limits negative career attitudes such as career indecision [66]. In this context, CO has been found to be related to the relationship between career adaptability and future career intentions [67], the relationship between mentoring and career adaptability and the relationship between mentoring and career self-efficacy [68], the relationship between variable career orientation and job search [69], the relationship between conscientiousness and neuroticism and career commitment [70], the relationship between impostor phenomenon and career satisfaction, and the relationship between impostor phenomenon and perceived career success [71]. On the other hand, career-choice anxiety and career-choice pessimism mediate the relationship between anxious attachment and career ambivalence [72]. In a similar example, career-choice pessimism and intrinsic motivation mediate the relationship between attachment and career adaptability [73]. Furthermore, Lerkkanen, Kivelä, Peterson and Sampson Jr [53], in their study investigating the extent to which career-related IB and career indecision influence career guidance needs, stated that more research is needed to examine the relationship between optimistic/pessimistic thinking styles and career indecision. In this context, it was noted that there is a gap in the literature regarding the mediating effect of CO in the relationship between employment-related IBs and CD. Therefore, the second hypothesis of the research is proposed as follows:

*H2*: *CO mediates the effect of women MIS students’ IBs related to employment on women’s CDs in the ICT sector*.

### Mediating role of PE

Perceived employability (PE) is a reflection of the internal dimension, in which the individual evaluates his/her own competencies for employment, and the external dimension, which consists of his/her perceptions of the labor market. In the intrinsic dimension, the individual evaluates factors of personal origin such as academic performance and employment skills. The external dimension includes individual perceptions of environmental factors such as labor market conditions, professional fields of study, university reputation, occupational demand and opportunities [74]. In the current labor market, possessing multiple skills such as flexibility, adaptability, and career self-management is considered crucial for career development. These abilities are also central to the PE phenomenon. In the modern working life where issues such as unemployment, job insecurity and mechanization come to the fore, PE plays a critical role for new entrants to employment. PE, which reflects a subjective assessment of an individual’s ability to compete in the labor market, covers all individual assessments and perceptions from the stage of finding a job to sustainable employment. In this regard, it mediates the relationships between psychological capital and school-to-work transition and career satisfaction [75]. PE also plays a mediating role in the relationship between core self-evaluations and preparatory job search behavior [76]. However, there is no existing literature on the mediating effect of PE in the relationship between employment-related IB and CD. The question of whether PE evaluations, especially towards STEM-based education and employment fields where women are less visible, play a mediating role in the relationship between IB and CD regarding employment constitutes a specific focus of the current research. For this reason, the third hypothesis of the study was established as follows:

*H*_*3*_: *The effect of women MIS students’ IBs related to employment on women’s CDs in the ICT sector is mediated by PE*.

### Serial multiple mediating role of CO and PE

The participation of women in employment is essential for the socio-economic development of countries due to various interactive benefits, such as promoting equality, harnessing human potential, generating economic growth, and reducing poverty [77]. Despite the ICT sector being viewed as a driver for increasing women’s employment [78], it is noteworthy that women’s representation in the sector remains relatively low [27]. A comprehensive analysis of the factors that affect this process can provide guidance for academic knowledge, practice, and policies on female labor force participation (FLFP) to help resolve this paradoxical situation. To this end, the present study also investigated the various effects of mediating variables to reveal more complex aspects of the relationship between women’s IBs and CDs towards employment and to gain a comprehensive understanding of the issue. In this context, testing whether the effect of IB on CD is mediated by more than one variable at the same time may open up different perspectives for promoting FLFP. Therefore, the fourth hypothesis of the study proposes to analyze the serial multiple mediation effect of CO and PE by creating a causal chain from IB to CD:

*H*_*4*_: *The effect of women MIS students’ IBs related to employment on women’s CDs in the ICT sector is serially mediated by CO and PE*.

## Materials and methods

### Sample and procedure

The participants of the study consisted of 232 women students studying at undergraduate level in 4-year faculties in the field of ICT in Turkey, who were determined by purposeful sampling method in accordance with the purpose of the study. In their 2021 study, Hair *et al*. [79] highlighted the suitability of the inverse square root method proposed by Kock and Hadaya [80] for calculating the minimum sample size. In accordance with this methodology, the lowest path coefficient within the model is of paramount importance in determining the requisite sample size. The proposed formula indicates that the square of the result obtained by dividing 2,486 by the lowest path coefficient at a 5% confidence level represents a determining criterion for the minimum sample size. Given that the lowest path coefficient in this study is 0.17, the minimum sample size is 213 participants. Consequently, it can be concluded that 232 participants constitute an adequate sample size for this study.

The age range of the participants was between 18 and 36, with an average age of 20.77 ± 2.11. The largest age group was 20 years old, with 52 participants (22.6%). In terms of grade levels, 74 participants (32.2%) were in the 3rd grade level, while the least number of participants, 36 (15.7%), were in the 4th grade level. This distribution aligns with the typical undergraduate age range of 18–24 years in Turkey. A small proportion of participants (3%, n = 7) were over the age of 30. Consequently, the sample is considered representative of the undergraduate population in ICT-related programs, with limited influence from older age groups.

Data for the study was collected on the online platform between 03/01/2024 and 29/02/2024. The questionnaire began with an informed consent form, ensuring voluntary participation. Participants who did not meet the criteria or declined to participate were excluded from the study.

### Ethical considerations

#### Institutional review board statement

The study was conducted according to the guidelines of the Declaration of Helsinki. In addition, the ethical approval of this study was obtained by the decision of Akdeniz University Social Sciences and Humanities Scientific Research and Publication Ethics Committee dated 25 December 2023 and numbered 569, and written consent was obtained from the sample who voluntarily participated in the study.

### Measures

This study examined four key dimensions: irrational beliefs about work employment (IB), career optimism (CO), perceived employability (PE) and career decisiveness (CD). The assessment of IB was conducted using the ’Career Anxiety’ scale, which was developed by Tsai, Hsu, and Hsu [52]. This scale consists of eight items and focuses on the career-related anxieties and irrational beliefs of the participants. The construct of CO was measured with a scale adapted from the “Career Futures Inventory”, which was developed by Rottinghaus, Day, and Borgen [55]. This scale assesses individuals’ positive perspectives towards their career. The construct of PE was assessed with a three-item scale adapted by Jackson and Tomlinson [57], which measures participants’ perception of their competitiveness in the labor market. The construct of CD was measured with a scale developed by Lounsbury, Hutchens, and Loveland [45], which examines participants’ degree of certainty and determination towards their career choices. The integration of these scales allows for a comprehensive examination of the impact of irrational beliefs about work employment (IB) on CD within the framework of complex relationships mediated by CO and PE.

Among these scales, only the "Career Anxiety" scale is a 4-point Likert scale, and the other scales are 5-point Likert scales. In addition, the reliability values of the scales in this study were 0.74, 0.82, 0.77, and 0.83, respectively. Therefore, it can be said that the reliability of the scales is high.

## Results

### Scale validation

Prior to testing the hypotheses concerning the serial mediation effect, a measurement model analysis was conducted to ensure measurement validity. The results of the four-factor scale model are presented in Table 1.

**Table 1:** Findings on the goodness of fit of the scale model.

Chi-square/df	RMSEA	SRMR	CFI	TLI
1.689	0.0.54	0.079	0.92	0.91

Table 1 shows that the IB, CO, PE, and CD subscales of the scale had a good fit, with average variance extracted (AVE) values of 0.52, 0.54, 0.52, and 0.51, respectively. The absence of multicollinearity between the scales was confirmed by analyzing the variance inflation factor (VIF) scores (ranging from 1.355 to 1.954).

### Scale correlations

According to the findings in Table 2, IB is negatively and significantly related to all other scales (-0.512, p<0.001; -0.302, p<0.001; -0.467, p<0.001). On the contrary, there were positive correlations between CO and PE (0.608, p<0.001), CO and CD (0.662, p<0.001) and PE and CD (0.515, p<0.001).

**Table 2:** Correlation analysis results between scales.

	Mean	SD	1	2	3	4
**IB**	2.08	0.51	-			
**CO**	3.38	0.61	-0.512**	-		
**PE**	3.56	0.78	-0.302**	0.608**	-	
**CD**	3.06	0.84	-0.467**	0.662**	0.515**	-

**Correlations are significant at the 0.01 level (two-tailed);

*Correlations are significant at the 0.05 level (two-tailed)

### Common Method Bias (CMB)

In order to prevent the potential influence of common method bias (CMB), a number of measures were implemented in the study. Firstly, the questionnaire was meticulously constructed and respondents were guaranteed anonymity in order to minimize the potential influence of social desirability on their responses. Furthermore, Harman’s one-factor test was employed to ascertain whether the data were attributable to a single factor. The results of Harman’s one-factor test indicated that the total variance explained was 26.46%, a figure that is considerably below 50%. Consequently, the findings demonstrated that common method bias was not a significant issue in this study.

### Hypothesis testing

For hypothesis testing, the PROCESS plug-in developed for SPSS software was utilized and the four steps of Baron and Kenny [81] were followed in examining the serial mediation effect model. This technique is commonly employed in the analysis of intricate relationships between dependent and independent variables, and is regarded as the most suitable for the objectives of our study. The PROCESS macro was selected over alternative techniques due to its efficacy in identifying serial mediation effects and detecting indirect effects between various variables. These steps also constitute the hypotheses of the study. According to the serial mediation effect model given in Fig 1 for our research question, the total effect (c value) of IB on CD was first analyzed to talk about the existence of serial mediation (*H*_*1*_). When *H*_*1*_ is tested, since it is seen that IB has a negative and significant effect on CD (β = -0.767, SE = 0.09, p < 0.001), hypothesis *H*_*1*_ is accepted.

**Fig 2:**
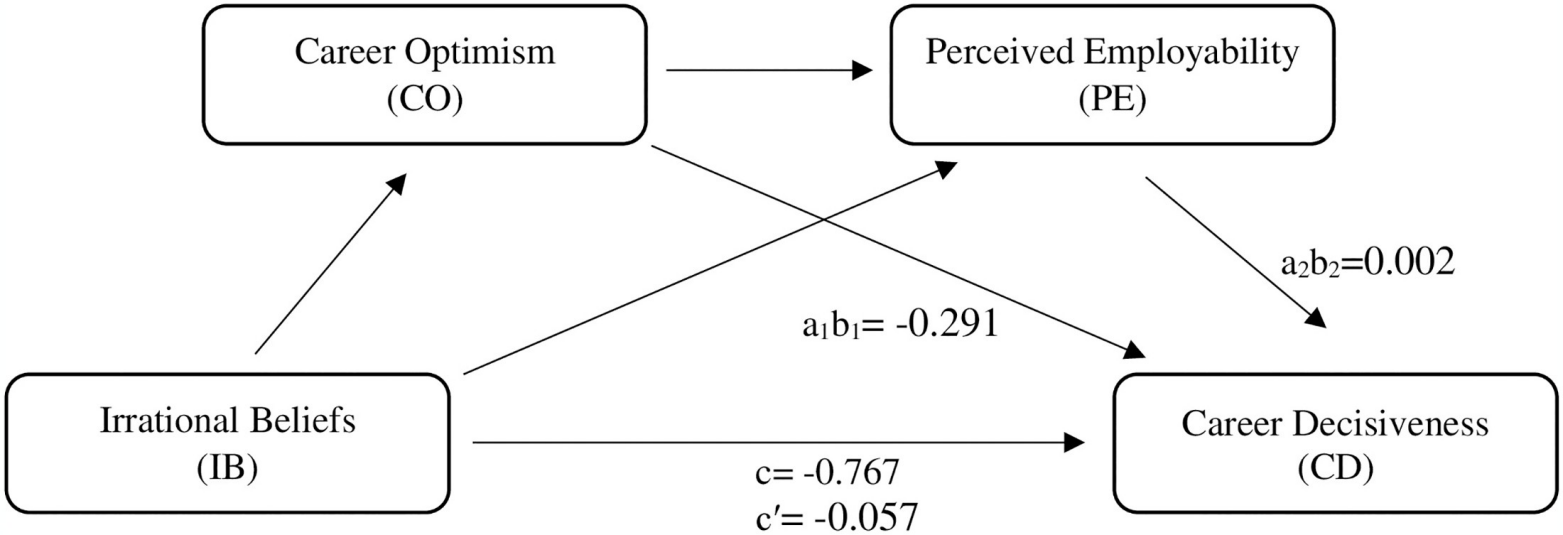
Multiple serial mediation model of the study with coefficients.

The study tested the mediating effect of CO on the relationship between IB and CD as per the second hypothesis. It was observed that IB had a negative indirect effect (α1b1 value) on CD through the mediation of CO (β = -0.291, SE = 0.04, p < 0.001), thus proving hypothesis *H*_*2*_. In another analysis (*H*_*3*_), in which the mediating role of the PE variable (α2b2 value) from the effect of IB on CD was examined, it was concluded that the PE variable had a non-significant indirect effect between these two variables (β = 0.002, SE = 0.01, p > 0.001) and the *H*_*3*_ hypothesis was rejected. According to *H*_*4*_, CO and PE variables have serial mediation effect on the effect of IB on CD. Accordingly, the indirect effect of IB on CD through serial mediation of these two variables was analyzed and it was found that this effect was negative and significant (β = -0.057, SE = 0.21, p < 0.001). Thus, hypothesis *H*_*4*_ was also accepted. As a result, all hypotheses except *H*_*3*_ hypothesis were proved and the summary table of the mediation effect is presented in Table 3. Furthermore, the coefficients of this mediation effect are illustrated in Fig 2.

**Table 3:** Mediation model summary.

Total Effect (IB → CD)	Direct Effect (IB → CD)	Relationship	Indirect Effect	Confidence Interval		t-statistics
-0.767 (0.000)	-0.289 (0.001)	IB → CO → PE → CD	-0.057	-0.168	-0.029	-2.656

## Discussion

This empirical research examines the manifestations of CD in the ICT context through the evaluations of women participants. Unlike previous studies in the related literature, it aims to understand the impact of women’s employment-related IBs on their CD when they are educated in a technology-oriented field through a serial multiple of CO and PE. The theoretical foundations of this research model are based on SCCT [32] and MST [33]. The study sheds light on women’s career-related attitudes that are formed based on their perceptual evaluations of individual and environmental factors. In this way, it provides insights for increasing women’s representation in the ICT sector in particular and in employment in general.

The analysis confirmed *H*_*1*_, indicating that women MIS students’ IBs related to employment negatively affect their CDs in the ICT sector. This finding is consistent with previous studies [82, 83]. Additionally, *H*_*2*_, which suggests that CO mediates the effect of IB on CD, was also confirmed. While there are findings in the related literature that suggest CO plays a role in the development of positive career attitudes, such as CD [48], no study has investigated this triple chain of relationships. Previous studies on employment-related IBs have focused on the relationship with self-efficacy [33, 76–78], one of the cognitive-person variables of the SCCT. At this point, Lerkkanen, Kivelä, Peterson and Sampson Jr [53] emphasized the importance and necessity of investigating optimism/pessimism tendencies to understand the relationship between career-related IBs and career ambivalence. In this sense, the confirmed *H*_*2*_ of the study contributes to fill the gap in the literature regarding the mediation of CO in the relationship between IB and CD. It can be concluded that employment-related IB indirectly affects CD through CO, which contributes to the development of a more stable career attitude.

Another hypothesis of the current study is the effect of subjective PEs, formed under the influence of cognitive, social, and environmental factors related to education and career orientation in technology-based fields where women are relatively underrepresented, on career attitudes. Bennett, *et al*. [84] concluded that women students are less confident in their ability to use digital technologies, learn new technologies, and interpret large data sets, especially in STEM fields. The study did not confirm *H*_*3*_, which suggests that PE mediates the effect of IB on CD. It was observed that women MIS students’ ratings of their PE had a non-significant indirect effect on the relationship between employment-oriented IB and CD. This finding is consistent with the results of Çelik [85]‘s study, which examined the predictive power of university students’ CO, hope, major suitability, and PEs on their career adaptability. In this context, it was determined that PE did not have a significant effect as a predictor variable when it was included in the regression model together with CO, departmental suitability and hope. When the relevant literature was examined, it was stated that there was no study related to this finding of the study. Contrary to expectations, it was thought that the fact that PE did not significantly predict career adjustment when it was included in the model with other predictor variables may be due to the negative meanings that university students attribute to their own employment skills. A PE that develops on the basis that they will assume a passive role in a limited environment may have the potential to complicate students’ career adaptability.

Conversely, Gerçek [86] discovered that career adaptability and self-PE mediated the relationship between career competencies and job search self-efficacy, respectively. This finding suggests that individuals who are proactive and adaptable in their careers may develop a stronger sense of employability. Therefore, it is believed that individuals who feel more employable may increase their confidence in their job search skills. According to this framework, individuals’ knowledge, skills, and abilities in their career fields can potentially facilitate career changes. Ahmed, *et al*. [87] also discovered that positive self-attributes, such as self-efficacy, influence career success that is mediated by PE. Lo Presti, *et al*. [88] found that employability orientation partially mediates the relationship between self-esteem and psychological well-being. Therefore, they suggest that self-esteem and employability orientation are personal resources that can have a positive impact on psychological well-being in the workplace.

In the final hypothesis, which is the specific contribution of the research to the literature, a two-mediator serial multiple mediation chain analysis was conducted in the relationship between employment-related IB and CD. The results showed that CO and PE have a serial mediation effect on the effect of women MIS students’ employment-related IB on their CD in the ICT sector. Therefore, *H*_*4*_ was confirmed. This finding provides insight into students’ career anxiety and attitudes towards their future careers. Employment-related IB constitutes one dimension of career anxiety [52]. Career anxiety can lead to negative career attitudes, such as career indecision [89–91]. Alrawadieh [92] found that perceived social support is less likely to alleviate the psychological distress and academic dissatisfaction of students experiencing employability anxiety. Jackson and Tomlinson [57] discovered that students’ engagement in career planning is motivated by favorable attitudes towards PE and a sense of career control. Raisi Sarteshnizi, *et al*. [93] confirmed the indirect effect of CO on PE through academic performance and job search self-efficacy. Rottinghaus, Day and Borgen [55] revealed that the perception of career futures is composed of career adaptability, CO, and knowledge dimensions. In his study, Çelik [85] found that CO was the strongest predictor of career adaptability. Additionally, Savickas [94] stated that career adaptability affects CD as a dimension of career futures. Correspondingly, career adaptability is effective on the career development decision in terms of individuals having the appropriate skills for their careers and their mastery of information about their careers.

Conversely, there are context-specific research findings that do not align with the assertion that women’s representation in ICT is underrepresented. For instance, Kurti et al. [95] contend that the prevailing discourses, which posit that women’s involvement in ICT education remains persistently low, fail to capture a universal and homogeneous phenomenon. In this context, the authors analyze the perceptions and motivations of female students in Sweden who are pursuing higher education in ICT-related fields. The researchers identify good employability and entrepreneurship opportunities as the primary motivating factors for women pursuing ICT-related higher education. Similarly, Corneliussen et al. [96] report that female high school students in Norway perceive technology as a means of contributing to society and view the ICT field as a remunerative employment opportunity and a potential avenue for career advancement. Hyrynsalmi and Hyrynsalmi [97] posit that a passion for technology serves as a motivating factor for adult Finnish women contemplating a career change in the software industry. Vergés Bosch et al. [98] highlight that the number of women employed in the ICT sector exceeds the number of women pursuing studies in ICT, indicating that a considerable proportion of women in ICT-related roles in Catalonia did not initially pursue engineering studies.

In their study investigating the persistence of women in STEM fields within four years after university graduation in the patriarchal social context of Kazakhstan, Kuchumova et al. [26] reveal that women in computer science and mathematics are exposed to a less gendered environment. In this context, the highest rates of persistence in STEM fields are observed among female graduates who have specialized in computing and mathematics. The findings of this study are consistent with those of previous research conducted in East Asian countries such as Malaysia, where the computing field is dominated by female employees. Mehta [99] highlights that women constitute a significant proportion of the workforce in the ICT sector in India, and that the sector as a whole is characterized by favorable working conditions for women. In this context, she posits that the sector predominantly employs young women from affluent socio-economic backgrounds, with high levels of education and from urban centers. Dlouhy and Froidevaux [20] investigate the career trajectories of professionals with STEM degrees from universities in Germany, Austria and Switzerland. Their findings indicate a higher proportion of female professionals in the non-STEM career model compared to those who have discontinued their studies. Furthermore, no differences in career patterns were identified among STEM graduates based on ethnic minority status. This is attributed to the German government’s policy of actively attracting foreign STEM professionals to Germany since 2005.

In addition to all these comprehensive discussions, it is thought that evaluating the findings regarding the mediating effect of CO and PE in the interaction between IB and CD through the lenses of SCCT and MST will contribute to the emergence of a more detailed approach to elucidating the dynamics that shape career decisions. Acceptance of hypothesis H_1_ revealed that irrational beliefs about employment have a negative and significant effect on career decisiveness. In other words, it has been determined that the individual’s fear of being involved in a new environment, being afraid of taking responsibility, not trusting the adequacy of his/her interpersonal communication skills, thinking that his/her university does not assist future employment opportunities, his/her family’s influence on his/her career choice, and his/her belief that he/she did not learn enough knowledge and skills at school [52] have negative effects on career decisiveness. This finding highlights the need for interventions aimed at addressing and reducing irrational beliefs about employment to increase career decisiveness. In reducing the negative effect of IB on CD, it can be stated that SCCT provides expansions based on self-efficacy, outcome expectations, and goals [32], while MST provides expansions based on capability and context beliefs as well as the role of goals and emotions [33]. Shu, Peng, and Wang [100] found that individuals with employment anxiety are guided by their emotions and act more intuitively when making career decisions. In a state of anxiety, the individual cannot make decisions on his/her own or ask for help from his/her immediate environment in making decisions. Efforts to prevent and alleviate employment anxiety can pave the way for more rational career decisions by allowing decisions to be made through cognitive analysis.

Acceptance of hypothesis H_2_ revealed that irrational beliefs about employment have a negative indirect effect on career decisiveness through career optimism. This finding suggests that irrational beliefs about employment may undermine career optimism, which in turn may affect career decisiveness. Wall [101] found that perceived supervisor support and social support significantly increased work engagement and career optimism played a critical mediating role in this relationship. Ogbuanya, Eseadi, Orji, Anyanwu, Joachim, and Out [102] found that rational emotive behavior therapy led to a significant reduction in students’ irrational career beliefs. The application of rational emotive behavior therapy in career counseling involves helping the client rationally confront irrational beliefs about career decision-making and replacing these beliefs with rational and useful ones. In this context, it is important to investigate whether applying rational emotive behavior therapy to students will lead to less anxiety about career decision-making and improvement in career self-efficacy beliefs. Davis and Turner [103] point out that revealing and reducing irrational beliefs and encouraging rational beliefs in the process of rational emotive behavior therapy facilitates the increase in self-determined motivation. Otu and Omeje [104] also stated that rational emotive career coaching significantly reduces dysfunctional career beliefs in newly graduated university students and that the effect of gender in this relationship should not be ignored. Smith, Caputi, and Crittenden [105] found that acceptance, one of the glass ceiling beliefs, was negatively associated with work engagement. Another glass ceiling belief, denial, is positively correlated with subjective success dimensions such as career satisfaction and work engagement. In this context, it can be said that considering the cognitive, emotional, behavioral, and motivational processes affecting the career decision together with their theoretical basis provides a solid foundation for reducing the individual’s irrational beliefs about career and increasing optimistic tendencies regarding his/her future career.

Hypothesis H_3_ was rejected because irrational beliefs about employment did not significantly affect career decisiveness through perceived employability. Duggal, Lim, Khatri, Thomas, and Shiva [106] emphasize that SCCT forms the cornerstone of self-perceived employability research. In this context, individuals are more successful in areas where they are more motivated and have strong efficacy beliefs and outcome expectations. Self-perceived employability, as a concrete reflection of outcome expectations, has significant effects on individuals’ career sustainability. An individual’s confidence in their abilities greatly increases their chances of being employed. In this context, self-perceived employability is a perception based on the evaluation of individual and contextual factors based on personality traits and cognitive processes. Bennett, Ananthram, Lindsay, Benati, and Jevons [107] suggest that students’ self and career exploration, including their views on whether and how their programs are linked to their future employment, impacts efficacy beliefs and outcome expectations. In the research conducted by Bennett, Knight, Bawa, and Dockery [108], it was found that female students had higher confidence in career decision-making, career identity, and career commitment than male students in both STEM and non-STEM student groups. This finding suggests that a uniform approach to STEM career decision-making based on gender differences is unlikely to be successful. At this point, comprehensive approaches are needed that address in detail the differences between STEM fields, non-STEM fields, and specific STEM fields, as well as gender-based differences.

Acceptance of hypothesis H_4_ revealed that the indirect effect of irrational beliefs about employment on career decisiveness was negative and significant through career optimism and perceived employability. This finding provides an important foundation for understanding how the relationship between IB and CD is mediated by individuals’ social, cognitive, and motivational evaluations of their career decisions. In other words, examining the mediating variables affecting the relationship between IB and CD reveals the multifaceted nature of employment barriers. This allows for a more detailed perspective to be presented to address these challenges. Bennett, Knight, Bawa, and Dockery [108] emphasize that students’ occupational engagement in STEM is influenced by increasing interest in STEM careers and societal perceptions that STEM careers are well-received, relatively stable, and personally rewarding. Jo, Park, and Song [109] point out that technology has both positive and negative effects on career competencies. In their research based on boundaryless career theory, Duggan, Sherman, Carbery, and McDonnell [110] found that algorithmic management practices in platform organizations have negative effects on career competencies by restricting employees’ abilities to navigate their roles and develop transferable competencies. In this context, Donald, Van der Heijden, and Baruch [111] suggest that the sustainable career perspective can be expanded by including various actors and contextual factors in the analysis of employability and career development research.

In social science studies, a single mediating variable may not completely explain the relationship between independent and dependent variables. Therefore, multiple mediation models provide a more comprehensive perspective. This allows for the evaluation of more than one mediation effect simultaneously. Overall, it is accepted that mediation analysis is a better approach to analyze and explain the complex model structures of studies dealing with multifaceted social phenomena [112]. With this approach the multiple mediation model proposed in this study contributes to the development of comprehensive insights into the factors that affect women’s career decisions in technology-oriented fields. Furthermore, the underrepresentation of women in STEM fields is not an isolated phenomenon; rather, it is part of a broader pattern observed in numerous sectors of the labor market. The multi-mediation model enables a more nuanced comprehension of the forces that inform women’s career decisions. Consequently, it provides a framework for the formulation of more efficacious strategies to address the issue of female representation in STEM and other sectors. In this context, it is thought that the four-variable structure and multiple mediation model of the current study can be transferred to industries such as aviation, space, finance, and investment management, where women are relatively less represented. Thus, by revealing the effects of individual and contextual factors that shape women’s career decisions in these fields, initiatives aimed at increasing women’s career stability can be supported.

## Conclusion

This study presents empirical evidence on the impact of employment-related IBs of women MIS students on their CD. The study contributes to the understanding of the role played by CO and PE as serial multiple mediators in limiting the negative effects of women’s employment-related IBs on CD in the ICT field. The findings reveal a negative aggregate effect of employment-related IBs on the CD of women MIS students in the ICT sector. Moreover, CO and PE mediate this relationship sequentially. These two mediated relationship chains, which have their theoretical foundations in SCCT [32] and MST [33], reveal the importance of subjective perceptual and attitudinal factors, including women students’ individual and contextual evaluations of the impact of their IBs on their CDs regarding employment in the ICT sector. Encouraging women students’ COs and PEs in a positive way may help to limit the negative effects of employment-oriented IBs on CD. This may pave the way for a more egalitarian labor market with career-oriented women employees.

The gender gap in employment, highlighted by worrying statistics, not only hinders women’s economic and social empowerment but also undermines sustainable development. Moreover, despite the increasing demand for STEM skills, women are still significantly underrepresented in these fields today. At this point, they face obstacles from cognitive beliefs, emotional arousal processes, and contextual understandings. Therefore, their career motivations are determined by both individual factors and interactions beyond their control. The current study highlights the need for multi-faceted approaches to elucidate the complex structures surrounding women’s career decisiveness attitudes in technology-related fields. It addresses the critical gap in understanding the factors affecting career decisiveness in the ICT sector, especially among female MIS students. It establishes a solid theoretical foundation by integrating complementary perspectives from major theories to fill this gap. Thus, it proposes a research model that reflects the effects of individual, work-related, organizational, and societal factors on women’s career decisiveness.

Analyzing the mediating effect of CO and PE in the interaction between employment-related IBs and CD highlights the cognitive, social, and motivational dimensions of career decision-making. This methodological approach adds depth to the research by showing how the variables examined affect career decisiveness. It suggests that individual and environmental factors are important for women’s employment outcomes. Understanding these factors can guide more effective interventions to help women build confidence in their careers and improve their career decision-making processes. It can also help assess how the complex relationships between these variables change and differ across contexts. As a result, this research serves as an important resource for promoting gender equality in employment and informing various stakeholders about the ongoing challenges and opportunities to achieve this goal. In this respect, it contributes to the academic understanding of women’s career decisiveness in the ICT sector, it also offers practical implications that can help create a more inclusive workforce in employment, economic growth, and sustainable development.

### Theoretical implications

The study’s theoretical significance is evident in its focus on investigating the factors that affect women’s CDs in the ICT sector through a multiple mediation model. This research extends the existing literature on career planning and management in HRM by demonstrating that women students’ employment-related IBs have a significant impact on their CDs. The study also provides guidance on how to promote FLFP, especially in technology-oriented fields, and increase women’s motivation for career determination. Additionally, it presents a more comprehensive perspective through a serial multiple mediation model that includes CO and PE variables in the analysis to reveal the impact of employment-related IBs on CD. There is no literature that analyses how these four variables work together. The study’s specific focus on women participants and the use of a two-mediator serial multiple mediation model analysis are strengths. Overall, this research provides a richer understanding of the interaction of various individual and contextual factors in women’s career determination.

### Practical implications

The research results contribute to a comprehensive understanding of ways to strengthen women’s career determination attitudes in the ICT sector and the labor market in general. It is important to explore the extent to which women’s career attitudes are shaped by their perceptual evaluations of individual and environmental factors for increasing the representation of women in employment. To encourage participation, it is essential to develop strategies that address specific individual, organizational, and contextual factors. Career development programs designed for students as potential actors in the labor market should include employment-oriented topics such as IB, CO, and PE. This can raise awareness of skills that promote CD among both educational institutions and employers. In addition, to promote women’s career determination, it is possible to design inclusive educational environments, implement mentoring programs, and offer support networks.

Cengiz [113] concluded that optimism plays a mediating role in the relationship between perceived social support and career determination among university students. The author suggests that organizing training and personal development programs for students who feel unsupported by their social environment may be beneficial. Kleine, *et al*. [114] found that career choice and development practices aimed at improving students’ career planning were effective in revealing positive effects on self-efficacy beliefs and reducing dysfunctional career-related concerns. According to Osborn, *et al*. [115], undergraduate career courses can effectively encourage career determination by reducing students’ dysfunctional career thoughts and increasing their career decision-making skills. Korkut Owen and Eraslan Capan [63] recommend implementing psychological counselling mechanisms in schools and organizing seminars to reduce students’ irrational career beliefs. Toyokawa and DeWald [116] emphasized the importance of career counsellors providing explanations about perceived career barriers during the process of guiding students’ career exploration and decision-making. Ebner’s [117] study revealed the effectiveness of internship programs in reducing career entry concerns and strengthening PE.

Jackson and Wilton [118] argue that universities must do more than just provide students with the skills needed to enter their chosen careers. They suggest that universities should also develop strategies to help students acquire career self-management skills in collaboration with industry. Additionally, it is crucial to establish policies that prevent employment discrimination based on social, cultural, political, economic, and technological norms. It is also important to implement workplace practices that facilitate the full integration of women into the workforce [119]. Implementation of these recommendations is expected to contribute to the achievement of the UN Sustainable Development Goals (SDGs), specifically SDGs 5 and 8, by promoting FLFP and strengthening human capital.

### Limitations and future research

This study has enriched the literature with its empirical findings on the factors that influence women’s attitudes toward CDs. There is no previous study in the relevant literature that examines how the variables included in the research model interact with each other. The current study has the potential to be of high original value to the relevant literature with its four-variable structure, the context it focuses on, and the results based on multiple mediation analysis. Although this study is important in terms of the new perspectives it offers to the literature with these strengths, it has some limitations that can be seen as opportunities for future research.

The study acknowledges limitations in its design and sampling and calls for further research to explore these aspects in different contexts to increase the generalizability of the findings. It should be noted that the sample size of this study is limited to that of Turkey, which could be considered a limitation. Furthermore, an analysis of the age distribution of the sample reveals a considerable age gap between the younger and older participants. In addition to the aforementioned limitation, it is possible to suggest that this situation may necessitate the examination of additional factors, such as experience and expectations, within the context of age-related differences in a subsequent study. The present study did not take into account the different grade levels involved in its analysis. It is plausible that students’ perceptions of IB, CO, PE, or CD may differ by grade level, given the potential discrepancies in academic experience and career readiness between first-year and senior students. Further research could examine the potential moderating influence of grade level on the relationships identified in this study. Moreover, future research is recommended to build on the mediation model found to be important in this study to further explore the explanation of women’s motivations for their CDs. The dimensions of this model could assist other researchers in designing experiments to gain comprehensive perspectives on women’s CDs. The study is cross-sectional, and longitudinal studies may be more useful in analyzing women’s motivations for career development at all stages. The study was designed using a quantitative approach. Future research can include qualitative studies to gain a deeper understanding of the factors influencing women’s attitudes towards CDs. Additionally, more comprehensive results can be obtained through mixed-methods studies that combine quantitative and qualitative approaches. The theoretical foundations of the current research model are based on SCCT [32] and MST [33]. In future studies, research models based on different theoretical backgrounds, such as Self-Determination Theory (SDT) [120], could be suggested to clarify women’s motivations for pursuing career development opportunities related to CD.

## Supporting information

S1 Data (ZIP)
