# Supplementary material for: Predicting women’s career decisiveness in the ICT sector: A serial multiple mediation model among MIS students
Source: PLoS One. 2024 Dec 20;19(12):e0316154. doi: 10.1371/journal.pone.0316154 (PMC11661593; doi:10.1371/journal.pone.0316154)
Supplement: S1 Data — (ZIP) [file pone.0316154.s001.zip › Questionairre Form - EN.pdf]

Career Decisiveness - Belief - Perception - Optimism

The Management Information Systems Department at Akdeniz University is seeking the input of its faculty members, namely Assoc. Prof. Dr. Nuray AKAR, Assoc. Prof. Dr. Tayfun YÖRÜK and Assoc. Prof. Dr. Ömür TOSUN, for a study investigating the factors affecting women's career determination. The questionnaire, which will be used as a data collection tool, comprises five sections and 28 questions and should take approximately 10 minutes to complete. The following information is provided regarding the data collected in the questionnaire:

1. Personal Identifying Information: It should be noted that the survey does not request any personal identifying information, such as name, surname, address or telephone number.

2. Digital Identifying Information: It should be noted that the completion of the questionnaire will not result in the collection of digital identifying information, such as IP address.

3. Data Sharing: The data collected from the survey will be used exclusively for research purposes. Following the anonymisation of the data, it may be shared with third-party organisations for the purpose of publishing the findings after analysis. The confidentiality of the participants' identities will be strictly maintained throughout the process.

Should you consent to participate in this study in light of the aforementioned information, you may proceed with the survey by selecting the "I agree" option provided below. It is our sincere hope that you will provide answers that are genuinely reflective of your opinions and that you will accept our gratitude for your participation.

* Specifies the mandatory question

*

- [x] I agree

Career Decisiveness - Belief - Perception - Optimism

Your grade level *

- 1
- 2
- 3
- 4

Age *

Your response

Do you have work experience in the field of Management Information Systems? If yes, please specify the duration. *

- No
- Yes

If your answer to the above question is ‘yes’, please specify the duration (e.g. 5 months, 3 years)

Your response

*

	1	2	3	4
I worry about future employment because I am afraid of a new environment.	○	○	○	○
I worry about future employment because I am afraid of taking on responsibilities.	○	○	○	○
I would worry about future employment if I was the breadwinner.	○	○	○	○
I worry about future employment because I do not have excellent interpersonal skills.	○	○	○	○
I worry about future employment because my university does not provide assistance for my future employment.	○	○	○	○
I worry about future employment because of scant job opportunities for my expertise.	○	○	○	○

I worry about future employment because my parents influence my career choice.	○	○	○	○
I worry about future employment because I have not learned sufficient knowledge and skills at school.	○	○	○	○

*

	1	2	3	4	5
I feel my skills and experiences will be sought after by future employers.	○	○	○	○	○
I believe that I will do well when competing with other graduates for jobs.	○	○	○	○	○
I feel confident to enter my targeted profession when I graduate.	○	○	○	○	○

*

	1	2	3	4	5
I get excited when I think about my career.	○	○	○	○	○
Thinking about my career inspires me.	○	○	○	○	○
Thinking about my career frustrates me.	○	○	○	○	○
It is difficult for me to set career goals.	○	○	○	○	○
It is difficult to relate my abilities to a specific career plan.	○	○	○	○	○
I understand my work-related interests.	○	○	○	○	○
I am eager to pursue my career dreams.	○	○	○	○	○
I am unsure of my future career success.	○	○	○	○	○
It is hard to discover the right career.	○	○	○	○	○
Planning my career is a natural activity.	○	○	○	○	○

Planning my career is a natural activity.	○	○	○	○	○

*

	1	2	3	4	5
I have made a definite decision about a career for myself.	○	○	○	○	○
I am having a difficult time choosing among different careers.	○	○	○	○	○
I am sure about what I eventually want to do for a living.	○	○	○	○	○
I know what kind of job I would like to have someday.	○	○	○	○	○
I am not sure what type of work I want to do when I get out of school.	○	○	○	○	○
I go back and forth on what career to go into.	○	○	○	○	○
