# Supplementary material for: Predicting women’s career decisiveness in the ICT sector: A serial multiple mediation model among MIS students
Source: PLoS One. 2024 Dec 20;19(12):e0316154. doi: 10.1371/journal.pone.0316154 (PMC11661593; doi:10.1371/journal.pone.0316154)
Supplement: S1 Data — (ZIP) [file pone.0316154.s001.zip › Questionairre Form - TR.pdf]

Kariyer Kararlılığı - İnanç - Algı - İyimserlik

Akdeniz Üniversitesi Yönetim Bilişim Sistemleri Ana Bilim Dalı Öğretim Üyeleri Doç. Dr. Nuray AKAR, Doç. Dr. Tayfun YÖRÜK ve Doç. Dr. Ömür TOSUN tarafından yürütülen "Kadınların kariyer kararlılığını etkileyen faktörlerin" araştırıldığı bu çalışmada görüşlerinize ihtiyaç duyulmaktadır. Veri toplama aracı olarak kullanılacak olan bu anket 5 bölüm ve 28 sorudan oluşmakta olup yaklaşık 10 dakika sürmektedir. Anketimizde topladığımız verilerle ilgili olarak aşağıdaki bilgileri dikkatinize sunmak isteriz:

1. Kişisel Tanımlayıcı Bilgiler: Anketimizde, ad, soyad, adres, telefon numarası gibi kişisel tanımlayıcı bilgiler kesinlikle istenmeyecektir.

2. Dijital Tanımlayıcı Bilgiler: Anketi doldururken, IP adresi gibi dijital tanımlayıcı bilgiler de toplanmayacaktır.

3. Verilerin Paylaşımı: Toplanan anket verileri yalnızca araştırma amacıyla kullanılacak ve veriler anonim hale getirildikten sonra, analizin ardından bulguların yayınlanabilmesi adına 3. parti kuruluşlarla paylaşılabilecektir. Bu süreçte, katılımcıların kimlikleri kesinlikle gizli kalacaktır.

Yukarıdaki bilgiler ışığında bu çalışmaya katılmayı kabul ediyorsanız aşağıda yer alan "**Kabul ediyorum**" kutucuğunu işaretleyerek ankete devam edebilirsiniz. Cevaplarınızı içtenlikle vereceğinizi umuyor, katılımınız için teşekkür ediyoruz.

* Zorunlu soruyu belirtir

*

- [x] Kabul ediyorum

Kariyer Kararlılığı - İnanç - Algı - İyimserlik

Sınıfınız *

- 1
- 2
- 3
- 4

Yaşınız *

Yanıtınız

Yönetim Bilişim Sistemleri alanında iş tecrübeniz var mı? Varsa süresini belirtiniz. *

- Yok
- Var

Yukarıdaki souya cevabınız "evet" ise süresini belirtiniz (Örn: 5 Ay, 3 Sene gibi)

Yanıtınız

*

	1	2	3	4
Yeni bir ortamın içine dahil olmaktan korktuğum için gelecekte iş bulma konusunda endişeliyim.	○	○	○	○
Gelecekteki işim konusunda endişeliyim çünkü sorumluluk almaktan korkuyorum.	○	○	○	○
Evin geçimini sağlayan kişi olsaydım gelecekteki işim konusunda endişelenirdim.	○	○	○	○
Gelecekteki çalışma hayatımla ilgili endişelerim var çünkü mükemmel insan ilişkileri becerilerine sahip değilim.	○	○	○	○
Gelecekteki işim konusunda endişeliyim çünkü üniversitem gelecekteki işim için yardım sağlamıyor.	○	○	○	○
Uzmanlığıma uygun iş fırsatlarının az olması nedeniyle gelecekteki işim konusunda endişeliyim.				

Gelecekteki işim konusunda endişeliyim çünkü ailem kariyer seçimimi etkiliyor.	○	○	○	○
Okulda yeterli bilgi ve beceri öğrenemediğim için gelecekte iş bulma konusunda endişeliyim.	○	○	○	○
Okulda yeterli bilgi ve beceri öğrenemediğim için gelecekte iş bulma konusunda endişeliyim.	○	○	○	○

*

	1	2	3	4	5
Gelecekteki işverenler tarafından becerilerimin ve deneyimlerimin talep edileceğini hissediyorum.	○	○	○	○	○
İş için diğer mezunlarla rekabet ederken başarılı olacağıma inanıyorum.	○	○	○	○	○
Mezun olduğumda hedeflediğim mesleğe girmek için kendime güveniyorum.	○	○	○	○	○

*

	1	2	3	4	5
Kariyerim hakkında düşündüğümde heyecanlanıyorum.	○	○	○	○	○
Kariyerim hakkında düşünmek bana ilham veriyor.	○	○	○	○	○
Kariyerimi düşünmek beni hayal kırıklığına uğratıyor.	○	○	○	○	○
Kariyer hedefleri belirlemek benim için zor.	○	○	○	○	○
Yeteneklerimi belirli bir kariyer planıyla ilişkilendirmek zor.	○	○	○	○	○
İşle ilgili ilgi alanlarımı anlayabiliyorum.	○	○	○	○	○
Kariyer hayallerimin peşinden gitmeye hevesliyim.	○	○	○	○	○
Gelecekteki mesleki başarımdan emin değilim.	○	○	○	○	○
Doğru kariyeri keşfetmek zordur.	○	○	○	○	○
Kariyerimi planlamak benim için doğal bir faaliyettir.	○	○	○	○	○

Kariyerimde kesinlikle doğru kararlar alacağım.	◯	◯	◯	◯	◯

*

	1	2	3	4	5
Kendi kariyerimle ilgili kesin bir karar verdim.	○	○	○	○	○
Farklı kariyerler arasında seçim yapmakta zorlanıyorum.	○	○	○	○	○
En nihayetinde yaşamak için ne yapmak istediğimden eminim.	○	○	○	○	○
Bir gün ne tür bir iş sahibi olmak istediğimi biliyorum.	○	○	○	○	○
Okulu bitirdiğimde ne tür bir iş yapmak istediğimden emin değilim.	○	○	○	○	○
Hangi kariyeri seçeceğim konusunda bir ileri bir geri gidiyorum.	○	○	○	○	○
