# Supplementary material for: Predicting women’s career decisiveness in the ICT sector: A serial multiple mediation model among MIS students
Source: PLoS One. 2024 Dec 20;19(12):e0316154. doi: 10.1371/journal.pone.0316154 (PMC11661593; doi:10.1371/journal.pone.0316154)
Supplement: S1 Data — (ZIP) [file pone.0316154.s001.zip › Explanation.docx]

Dear Editor,

Please find attached the data file used for our analyses, as well as the original Turkish version of the survey questions and the translated version into English. It should be noted that the initial item in the questionnaire pertains to consent for participation in the survey. In this question, clarifications regarding data privacy and data sharing for the purposes of survey participation were provided. Considering these explanations, the participant was asked to consent to the utilization of the survey data. In the event that consent is not provided, the survey cannot be continued, and the participant is free to withdraw from the survey at any time.
